# What underlies sex differences in heart failure onset within the first year after a first myocardial infarction?

**DOI:** 10.3389/fcvm.2023.1290375

**Published:** 2024-01-23

**Authors:** Simon Leboube, Louise Camboulives, Thomas Bochaton, Camille Amaz, Cyrille Bergerot, Mikhail Altman, Thomas Loppinet, Maelle Cherpaz, Thierry Monsec, Catherine Sportouch, Annie Trinh, Camille Soulier, Anne Bernard, Genevieve Derumeaux, Nathan Mewton, Michel Ovize, Hélène Thibault

**Affiliations:** ^1^ Explorations Fonctionnelles Cardiovasculaires, Hôpital Louis Pradel, Hospices Civils de Lyon, Bron, France; ^2^ Laboratoire CarMeN – IRIS Team, INSERM, INRA, Université Claude Bernard Lyon-1, 21 Univ-Lyon, Bron, France; ^3^ Centre d'investigation clinique de Lyon, Hospices Civils de Lyon, Lyon, France; ^4^ Service de Cardiologie, Centre Hospitalier de Valence, Valence, France; ^5^ Service de Cardiologie, Clinique du Millénaire, Montpellier, France; ^6^ Service de Cardiologie, Hôpitaux Universitaires de Strasbourg, Strasbourg, France; ^7^ Service de Cardiologie, CHU de Nîmes, France; ^8^ Service de Cardiologie, Centre Hospitalier Universitaire de Tours, Tours, France; ^9^ Service de Cardiologie, Hôpitaux Universitaires Henri Mondor, Créteil, France

**Keywords:** echocardiography, sex differences, myocardial infarction, women, heart failure

## Abstract

**Background:**

Women are more likely to develop heart failure (HF) after myocardial infarction. However, diagnosis and reperfusion are often delayed.

**Objectives:**

To compare the prevalence of HF after primary percutaneous coronary intervention (PPCI)-treated ST segment myocardial infarction (STEMI) between sexes and to study its associations with comorbidities, infarct size, and left ventricular (LV) systolic and diastolic dysfunctions (DD).

**Methods:**

The patients with PPCI-treated anterior STEMI, from the CIRCUS study cohort, were followed up for 1 year and HF events were recorded. Evaluation of ejection fraction (LVEF) and DD were performed at baseline and at 1 year. The elevated LV filling pressure (LVFP) included Grades 2 and 3 DD.

**Results:**

Of the 791 patients from the CIRCUS study, 135 were women. At 1 year, the proportion of patients who developed HF was 21% among men and 34% among women (*p* = 0.001). In the subset of 407 patients with available diastolic parameters, the rate of HF was also higher in women. HF during the initial hospitalization was comparable between the sexes. However, women had a higher incidence of rehospitalization for HF within the first year after STEMI (14.1% vs. 4.1%, *p* = 0.005). Women were older with a higher prevalence of hypertension. The infarct size and LVEF were similar between the sexes. Elevated LVFP was observed more frequently in women than in men during the initial hospitalization and at 1 year (26% vs. 12%, *p* = 0.04, and 22% vs. 12%, *p* = 0.006, respectively). Interestingly, only initial elevated LVFP (HR 5.9, 95% CI: 2.4–14.5, *p* < 0.001), age, and hypertension were independently associated with rehospitalization for HF.

**Conclusions:**

After PPCI-treated anterior STEMI, despite comparable infarct size and LVEF, women presented a higher proportion of rehospitalization for HF than men. That was likely due to a greater DD associated with older age and hypertension.

## Introduction

Heart disease is the leading cause of death among women (1). In the setting of coronary artery disease (CAD), the awareness of the risk among women is low and sex disparity in the management of CAD is well established, with women being less likely to receive recommended diagnostic testing and therapies (2, 3).

In recent registries, women represent between 20% and 35% of patients with acute ST segment elevation myocardial infarction (STEMI) (4–6). The women are generally older, more often hypertensive, and have a worse prognosis than men with a higher risk of developing heart failure (HF) after an acute myocardial infarction (7–9). However, STEMI diagnosis and reperfusion are often delayed in women suggesting that this may lead to larger myocardial damage, worse systolic function, and subsequently higher risk of developing HF (10, 11). Therefore, we questioned whether this sex difference in prognosis after STEMI may be driven by either larger myocardial damage and left ventricular (LV) systolic dysfunction, or diastolic dysfunction and associated older age and comorbidities.

The aim of our study was (1) to confirm the increased proportion of HF in women after STEMI, even in the case of early reperfusion, and recommended medical treatment, and (2) to determine the factors independently associated with HF.

## Methods

We used the study population of the prospective, controlled, multicenter CIRCUS trial that enrolled patients with primary percutaneous coronary intervention (PPCI) for an anterior STEMI (12) who benefit from an early reperfusion and were followed up for 1 year. The patients were well characterized. In a subgroup of patients, a careful assessment of their LV systolic and diastolic functions was performed both immediately after STEMI and at 1 year.

### Study population

The CIRCUS trial enrolled 970 patients. The complete study protocol and primary results from this study have previously been published (12, 13). The study included patients presenting with acute STEMI (defined by clinical symptoms consistent with acute coronary syndrome associated with ST segment elevation of 0.2 mV or more in two contiguous anterior leads), within 12 h after chest pain onset and with an occluded left anterior descending coronary artery [thrombolysis in myocardial infarction (TIMI) flow grade of ≤1 at diagnostic coronary angiography]. All patients underwent percutaneous coronary intervention (PCI) and were treated according to the updated STEMI European guidelines. All subjects gave their informed consent before inclusion into the study (NCT01502774). The coronary angiographic data were analyzed by a central core laboratory. The area at risk according to angiography was determined as previously described (13). The total creatine kinase level, a surrogate for infarct size, was measured locally at admission and 4, 12, and 24 h after reperfusion.

Because the CIRCUS study treatment (cyclosporine) had no impact on heart failure or cardiac remodeling (12), we considered the whole study population as one single cohort of anterior STEMI patients. We assessed the left ventricular systolic and diastolic functions in all the patients with echocardiographic images available at baseline and 1 year after STEMI. The patients were divided into two groups according to their sexes: women and men. We then compared their clinical characteristics, echocardiographic assessments of both systolic and diastolic functions at baseline, and 1-year follow-up (FU) and clinical events.

### Echocardiographic assessment

Two-dimensional echocardiography was performed during the initial hospitalization (median duration: 4 days) and at 1-year of FU. Echocardiographic data were stored digitally and sent by all study centers to the central core laboratory of the Cardiology Department of our institution (Hospices Civils de Lyon) for analysis. The LV-centered apical four- and two-chamber views were systematically recorded for evaluation of left ventricular end-diastolic volume (LVEDV), left ventricular end-systolic volume (LVESV), and left ventricular ejection fraction (LVEF). Adverse left ventricular remodeling was defined as an increase of more than 15% of LVEDV between the two echocardiograms (baseline and 12 months). A first analysis of these three criteria (LVEDV, LVESV, and LVEF) was performed by expert readers according to Simpson's biplane rule.

LV diastolic parameters were measured to assess the grade of diastolic function. All measurements and analysis of the diastolic function were performed offline using an Echo PAC Clinical Workstation by expert readers blinded to all other clinical data including sex. Early mitral inflow velocity (E); late mitral inflow velocity (A); and mitral E wave deceleration time (VM DT) were measured from the pulsed Doppler mitral inflow obtained in the apical four-chamber view at the tips of the mitral valve leaflets. Lateral and septal tissue Doppler early diastolic mitral annular velocities (e’) were collected. The left atrium (LA) maximum volume was obtained in the apical four- and two-chamber views at end systole just before mitral valve opening. The pulmonary artery systolic pressure (PASP) was estimated by continuous wave (CW) Doppler of the tricuspid regurgitation (TR) jet velocity measured in apical four-chamber view with color flow imaging used for the optimization of CW Doppler with blood flow. Based on these measurements, the various ratios required to assess LV filling pressure (LVFP) were calculated: E/A, lateral E/e’ ratio, septal E/e’ ratio, average E/e’ ratio, and LA maximum volume indexed to body surface area. The LV diastolic function of each patient was then classified according to the ASE/EACVI 2016 guidelines algorithm for estimation of LV filling pressures (14). When it was possible to determine DD grade, patients were classified into 2 groups: (1) the “normal LVFP” group, which encompassed all patients with Grade 1 DD, and (2) the “elevated LVFP” group, which included Grades 2 and 3 DD.

### Clinical outcomes

The patients were followed clinically and contacted by phone 1, 3, 6, and 12 months after the index STEMI. All adverse clinical events were reviewed by a centralized event validation committee.

During the index hospitalization, “initial heart failure worsening” was defined as new symptoms or worsening of initial symptoms of HF (as noticed at the time of admission) requiring administration of intravenous diuretics. During the 1 year of follow-up, “rehospitalization for heart failure” was validated if the patient was admitted to the hospital with a diagnosis of HF. All HF events have been previously reported and validated (13). “Heart failure” corresponded to the association of HF events: initial HF worsening and/or “rehospitalization for HF.”

### Statistical analysis

Quantitative variables were described by sex and expressed as mean ± standard deviation. Categorical variables were expressed as numbers (percentage). Bivariate comparisons were made using Student's *t*-test for continuous variables or the Wilcoxon test when assumptions for normality were not validated. Χ^2^ tests (or Fisher's exact test when appropriate) were used for categorical variables.

Survival curves were constructed using the Kaplan–Meier method. The comparison between groups was conducted using the Log-rank test.

The determinants of rehospitalization for HF were evaluated using the Cox regression analysis. A multivariable model was estimated, including all univariate determinants at *p* < 10% or deemed to be clinically relevant.

The statistical testing was done at the two tests’ α level of 0.05. Data were analyzed using the R statistical software version 3.3.3.

## Results

### Heart failure outcome according to sex in the CIRCUS Cohort

**Figure 1:**
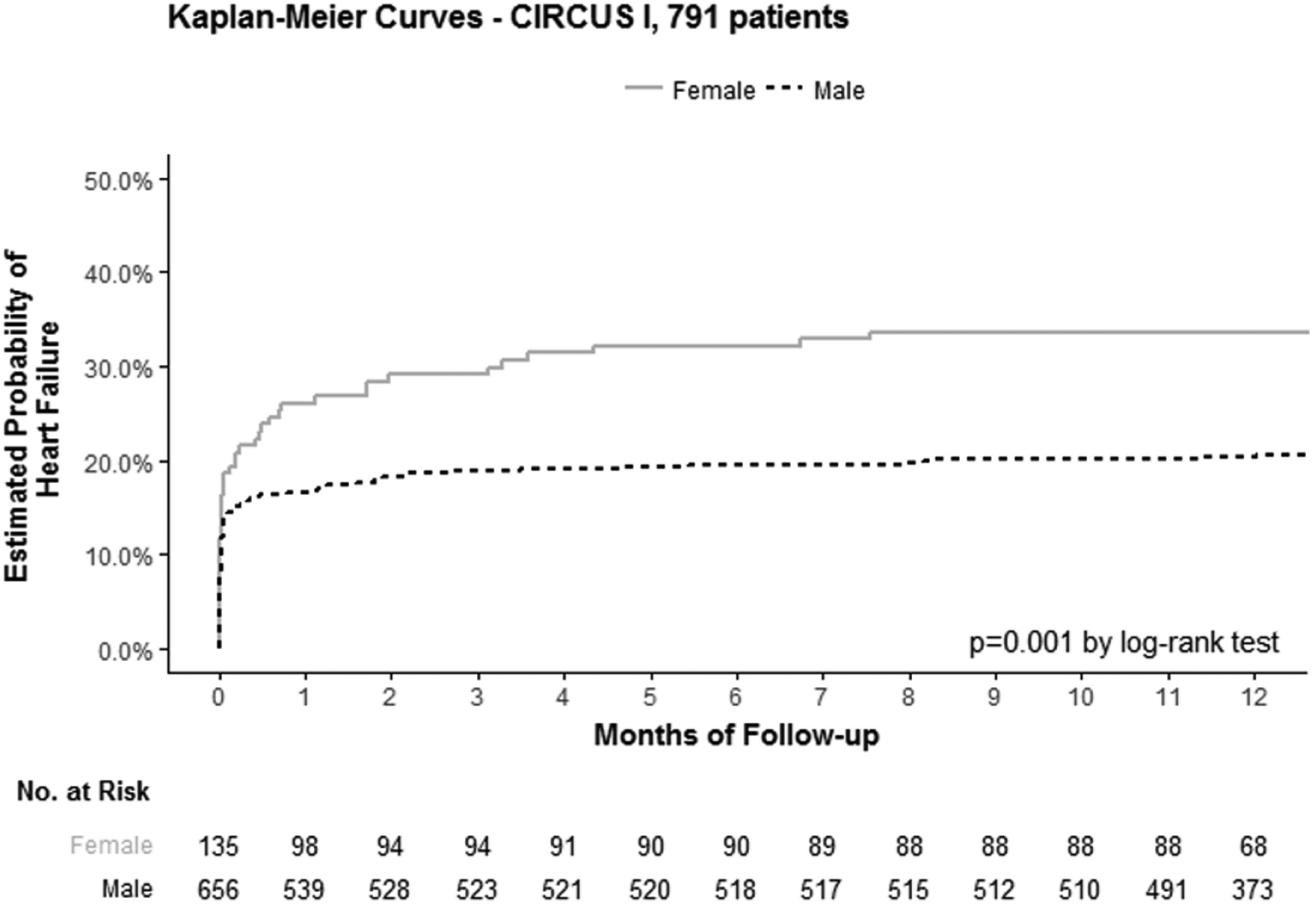
Kaplan–Meier curves of the estimated probability of heart failure according to sex (women: continuous line; men: dotted line), in the 791 patients included in the CIRCUS study analysis.

**Figure 2:**
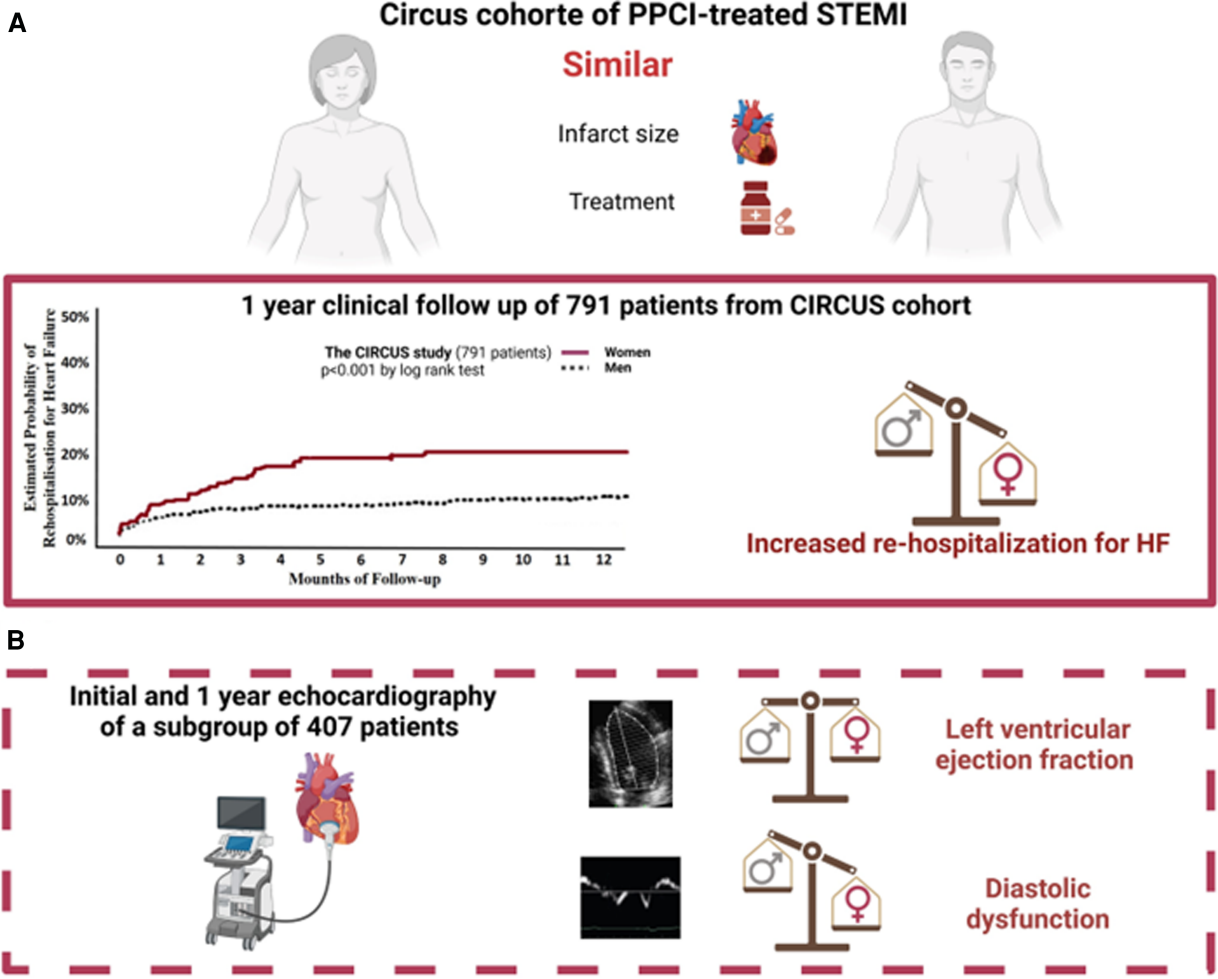
Graphical abstract. In this article, we reported a higher proportion of rehospitalization for heart failure in women than men in the complete CIRCUS study of 791 patients (**A**). We then studied a subgroup of 407 patients with echocardiographic analysis of systolic and diastolic functions available initially and at 1 year (**B**).

A total of 791 patients were included in the CIRCUS study analysis, including 82% men and 17% women. The proportion of heart failure (worsened HF during the initial hospitalization or rehospitalization for HF) was 34% in women vs. 21% in men (*p* = 0.001; Figure 1). This difference among women and men was even more pronounced for rehospitalization for HF: 19% among women vs. 9% among men (*p* < 0.001; Figure 2A).

### Systolic and diastolic function in the subgroup

Altogether 407 of the 791 initial patients, including 64 (15.7%) women and 343 (84.3%) men, had echocardiographic images available for assessment of both systolic and diastolic functions at baseline and 1 year after STEMI (Figure 2B).

#### Baseline characteristics

Baseline characteristics of this subpopulation were comparable to that of the whole study population. Cardiovascular risk factors and medical history were balanced between women and men (Table 1). However, women were significantly older (63.1 vs. 57.8 years, *p* < 0.008), more frequently hypertensive (50% vs. 28.6%, *p* = 0.001), and had a lower glomerular filtration rate (GFR) than men on admission. Total ischemic time and coronary artery status were similar between the two groups (Table 2). Women and men received similar pre-hospital medical care as well as comparable coronary intervention. In-hospital treatment of HF, including intravenous diuretics and non-invasive positive pressure ventilation, was similar between women and men (Supplementary Appendix Table S1). The area at risk and infarct size, as estimated by the creatine kinase peak and AUC, were not significantly different between the sexes (Table 2). The average in-hospital length of stay was slightly but significantly longer in women than in men (7.5 ± 4.0 vs. 6.6 ± 3.9 days, *p* = 0.025). Medical treatment at discharge was similar between the two groups (Supplementary Appendix Table S2).

**Table 1:** Baseline characteristics.

	Study population *n* = 407	Female *n* = 64	Male *n* = 343	*p*
Age	58.6 ± 12.2	63.1 ± 14.7	57.8 ± 11.5	0.008
BMI	26.5 ± 3.7	25.5 ± 4.5	26.7 ± 3.5	0.06
Current smoking	185 (45.5%)	27 (42.2%)	158 (46.1%)	0.664
Hypertension	130 (31.9%)	32 (50%)	98 (28.6%)	0.001
Diabetes mellitus	48 (11.8%)	6 (9.4%)	42 (12.2%)	0.658
Dyslipidemia	152 (37.3%)	22 (34.4%)	130 (37.9%)	0.693
Previous MI	16 (3.9%)	2 (3.1%)	14 (4.1%)	1
Previous coronary artery disease treated with PCI	18/21 (85.7%)	2/2 (100%)	16/19 (84.2%)	1
Multivessel disease	147/407 (36%)	21/64 (33%)	126/343 (37%)	0.647
Previous HF	0 (0%)	0 (0%)	0 (0%)	–
GFR (ml/min/1.73 m^2^)	100 ± 34	84 ± 34	103 ± 34	<0.001

Data are presented as no./total no. (%). Age is measured in years and BMI is measured in kg/m^2^.

**Table 2:** Pre-hospital management and angiographic data.

	Study population *n* = 407	Female *n* = 64	Male *n* = 343	*p*
Time from symptom onset to hospital arrival (min)	201 ± 171	214 ± 133	198 ± 176	0.07
Time from admission to treatment	62 ± 85	55 ± 47	63 ± 90	0.87
Total ischemic time (min)	263 ± 173	264 ± 138	263 ± 178	0.24
Killip class at admission				
1	327/371 (88.1%)	50/59 (84.7%)	277/312 (88.8%)	
>1	44/371 (11.9%)	9/59 (15.3%)	35/312 (11.2%)	
Infarct size				
Peak CK	3,986 ± 2,562	3,565 ± 2,103	4,063 ± 2,633	0.3
CK AUC	65,898 ± 37,298	57,674 ± 28,251	67,170 ± 38,410	0.36
Medication from first medical care to PCI				
Heparin	345/406 (85%)	60/64 (93.8%)	285/342 (83.3%)	0.05
GP IIb/IIIa inhibitor	174/406 (42.9%)	29/64 (45.3%)	145/342 (42.4%)	0.76
Loading dose of P2Y12 inhibitor	353/406 (86.9%)	54/64 (84.4%)	299/342 (87.4%)	0.64
Aspirin	375/406 (92.4%)	57/64 (89.1%)	318/342 (93%)	0.30
Morphine	232/406 (57.1%)	36/64 (56.2%)	196/342 (57.3%)	0.98
Site of LAD occlusion				0.55
Proximal or main	174 (42.8%)	30 (46.9%)	144 (42%)	
Medial/distal LAD or diagonal branch	233 (57.2%)	34 (53.1%)	199 (58%)	
Area at risk (%)	36.5 ± 8.4	36.9 ± 8.3	36.4 ± 8.4	0.86
TIMI flow grade before PCI				0.53
0	314/392 (80.1%)	53/62 (85.5%)	261/330 (79.1%)	
1	50/392 (12.8%)	5/62 (8.1%)	45/330 (13.6%)	
2	22/392 (5.6%)	4/62 (6.5%)	18/330 (5.5%)	
3	6/392 (1.5%)	0/62 (0%)	6/330 (1.8%)	
Thrombus aspiration	323 (79.4%)	49 (76.6%)	274 (79.9%)	0.66
Direct stenting	238/392 (60.7%)	31/62 (50%)	207/330 (62.7%)	0.08
Stenting	357 (87.7%)	51 (79.7%)	306 (89.2%)	0.05
No reflow observed on angiography	18 (4.4%)	4 (6.2%)	14 (4.1%)	0.50
TIMI flow grade after PCI				0.38
0	4/402 (1%)	1/64 (1.6%)	3/338 (0.9%)	
1	4/402 (1%)	1/64 (1.6%)	3/338 (0.9%)	
2	25/402 (6.2%)	2/64 (3.1%)	23/338 (6.8%)	
3	369/402 (91%)	60/64 (93.8%)	309/338 (91.4%)	

CK, creatinine kinase in UI/L; GP, GlycoProtein.

Data are presented as no./total no. (%). Times are represented as median ± standard deviation. Biological data are represented as absolute numbers. Times are in minutes. Area at risk in %.

#### Echocardiographic assessment

**Figure 3:**
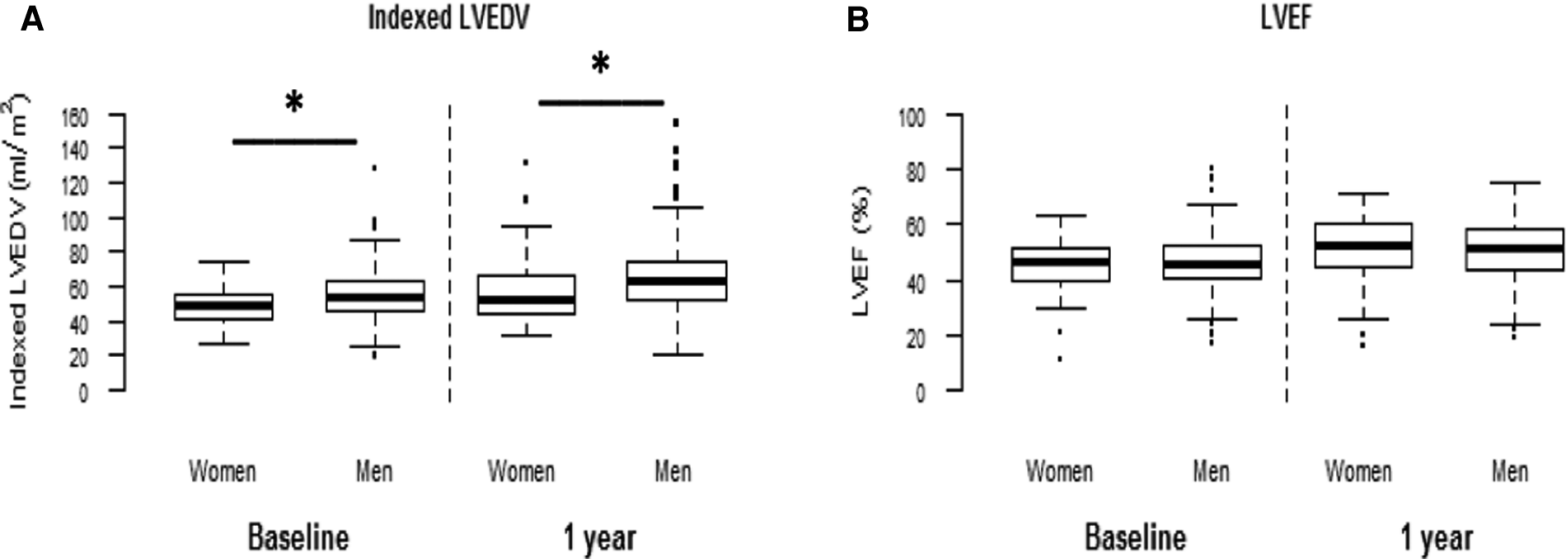
Left ventricular volumes and ejection fraction according to sex. Left ventricular volumes and ejection fraction were assessed by echocardiography (Simpson method) both at baseline and 1 year. *p < 0.002, between the two sexes.

Both at baseline and 1-year follow-up women displayed lower indexed LVEDV and LVESV than men (28.5 ± 18.6 vs. 30 ± 10.6 ml/m^2^ for LVEDV, *p* = 0.01, and 29.9 ± 16.9 vs. 33.7 ± 16.8 ml/m^2^, respectively, for LVESV; *p* = 0.02) (Figure 3). In both groups, LV volumes increased between baseline and 1 year. There was no difference in the increase in LV volumes or the percentage of adverse LV remodeling between men and women (44.4% vs. 48.5%, respectively; *p* = 0.64) (Supplementary Appendix Table S3). LVEF was similar in the two groups both initially and at 1 year of follow-up (45.4 ± 9.5% in women vs. 46.4 ± 8.8% in men, *p* = 0.74, and 51.0 ± 11.9 vs. 50.7 ± 10.1%, respectively; *p* = 0.55; Figure 3).

Peak E and A velocities were significantly greater in women than in men, both initially and at 1 year (Supplementary Appendix Table S4). However, E/A ratios were similar between women and men. At baseline, lateral tissue Doppler peak e’ was smaller in women than in men. Women had more often an E/e’ ratio above 14 at baseline (23.5% vs. 8%, *p* = 0.01) but not at 1-year follow-up (13.3% vs. 4.7%, *p* = 0.07). The TR jet velocity was higher in women than in men, both at baseline and 1-year follow-up. The indexed left atrium maximal volumes were not significantly different between the sexes (Supplementary Appendix Table S4). However, there was a trend toward higher volume in women than in men at 1-year follow-up (43.1 ± 14.0 ml/m^2^ vs. 39.2 ± 13.8; *p* = 0.068).

**Figure 4:**
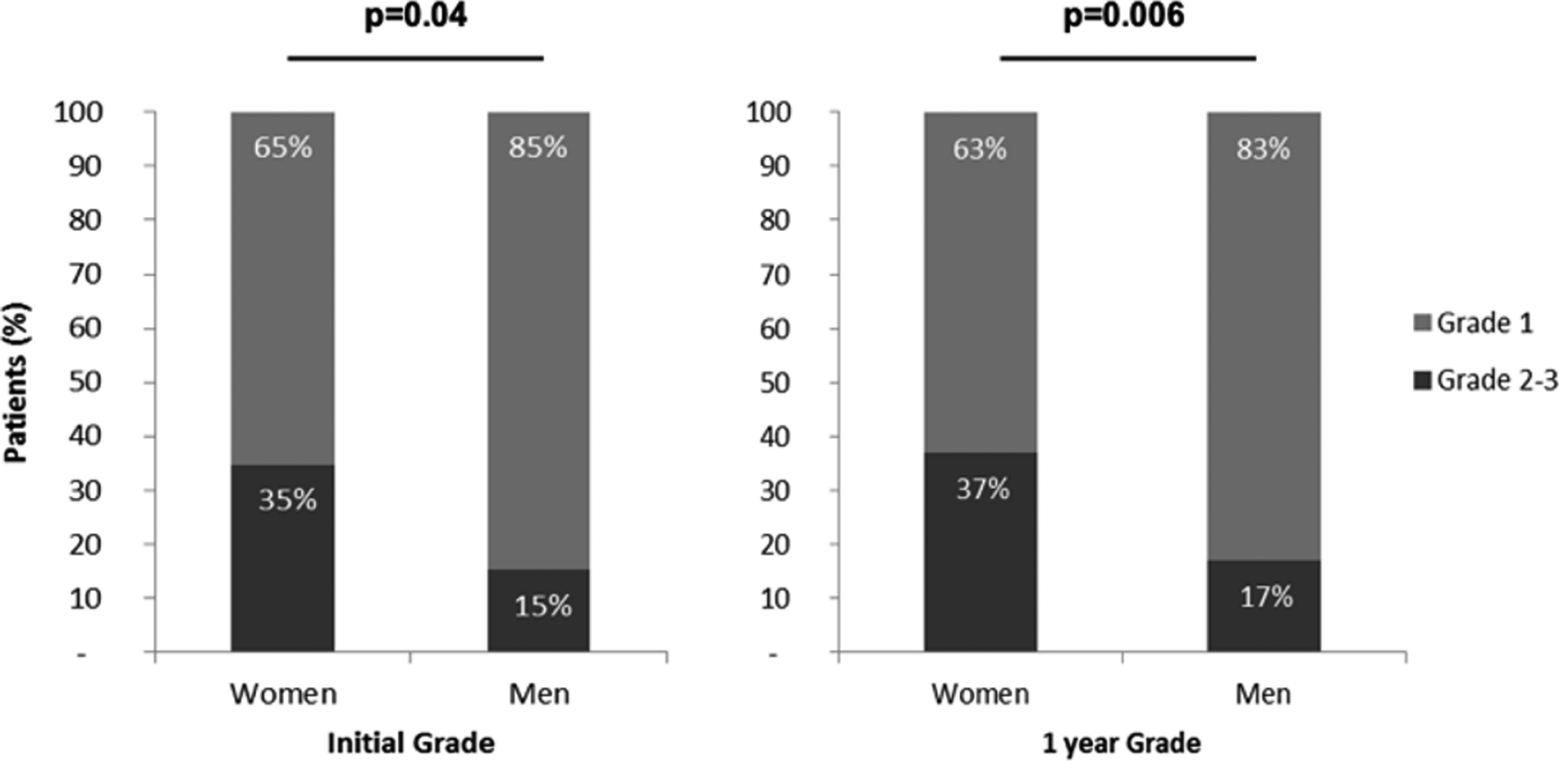
Comparison of diastolic dysfunction grade between women and men. Grades of diastolic dysfunction were evaluated by echography according to current guidelines (14). The “normal left ventricular filling pressure” group encompassed patients with Grade 1 diastolic dysfunction (light blue). The “elevated left ventricular filling pressure” group included Grades 2 and 3 (dark blue). Both at baseline and at 1-year follow-up, elevated LVFP was more frequent in women than in men.

Elevated LVFP (Grades 2 and 3 of DD) were more frequent in women than in men, both at baseline and at 1 year, (35% vs. 15%, *p* = 0.04%, and 37% vs. 17%, *p* = 0.006, respectively) (Figure 4).

#### Clinical outcomes

**Figure 5:**
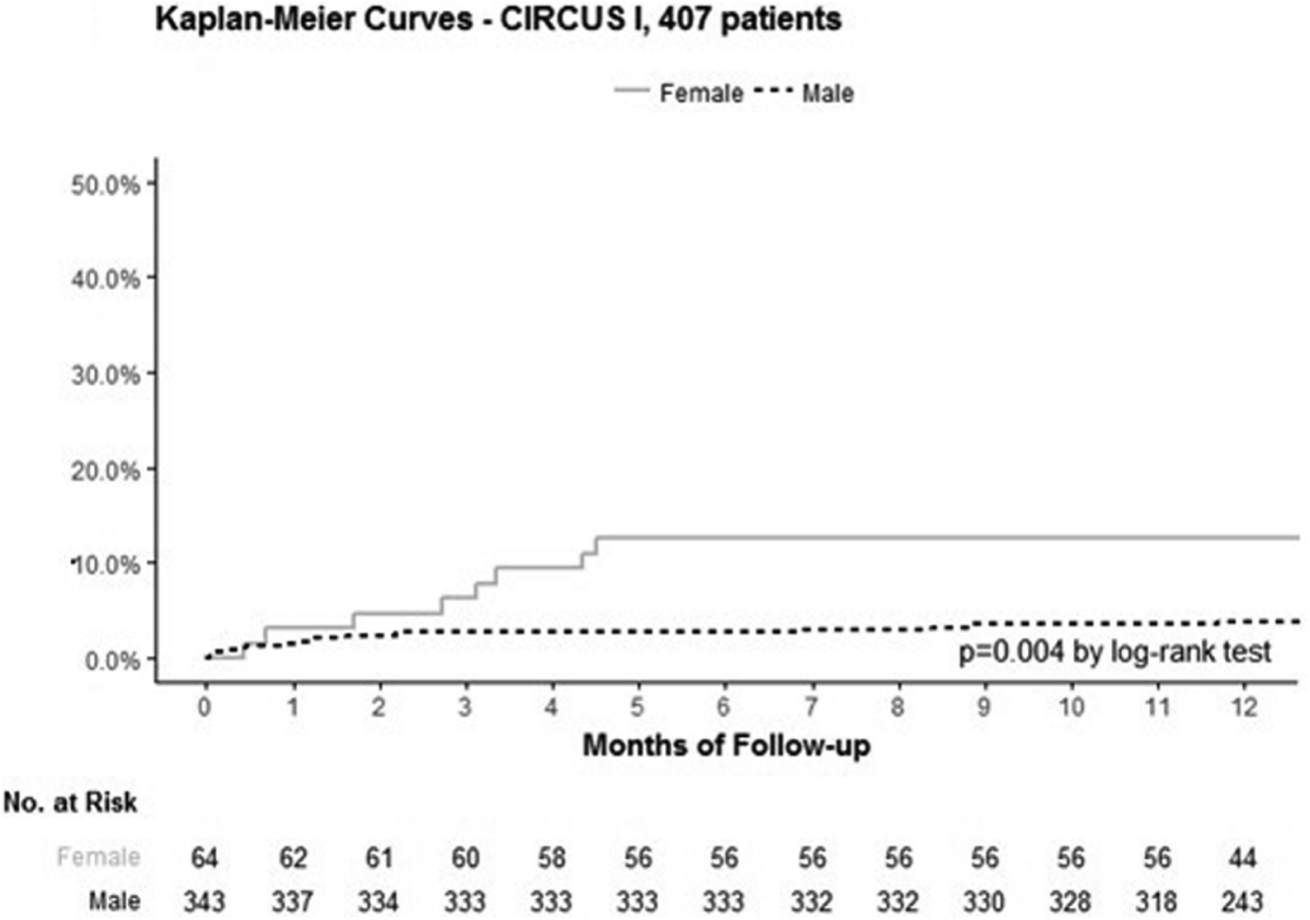
Kaplan–Meier curves representing the estimated probability of rehospitalization for heart failure according to sex in the 407 patients’ subgroup. Kaplan–Meier curves of the estimated probability of rehospitalization for HF in female (continuous line) and male patients (dotted line) in the 407 patients with echographic assessment of systolic and diastolic functions.

As for the whole study population, the incidence of HF was higher in women than in men in the subset of patients (20.3% vs. 10.8%, respectively; *p* = 0.05; Table 3). It is important to note that the worsening of HF during the initial hospitalization was comparable between the sexes (10.9 vs. 8.2%, *p* = 0.62). But, women had a significantly higher rate of rehospitalization for HF than men within the first year after STEMI (14.1% vs. 4.1%, *p* = 0.005; Figure 5). Death from any cause, cardiovascular death or recurrent myocardial infarction (MI), were rare and similar in both groups during the 1-year follow-up (Table 3).

**Table 3:** Clinical outcomes.

	Study population *n* = 407	Female *n* = 64	Male *n* = 343	*p*
Heart failure	50 (12.3%)	13 (20.3%)	37 (10.8%)	0.05
Worsening of HF	35/407 (8.6%)	7/64 (10.9%)	28/343 (8.2%)	0.62
Rehospitalization for HF	23/407 (5.7%)	9/64 (14.1%)	14/343 (4.1%)	0.005
Other outcomes				
Death from any cause	1 (0.2%)	0 (0%)	1 (0.3%)	0.99
Cardiovascular death	1 (0.2%)	0 (0%)	1 (0.3%)	0.99
Recurrent myocardial infarction	13 (3.2%)	2 (3.1%)	11 (3.2%)	0.99
Stroke	7 (1.7%)	1 (1.6%)	6 (1.7%)	0.99
Cardiogenic shock	7 (1.7%)	0 (0%)	7 (2%)	0.60
Unstable angina	9 (2.2%)	2 (3.1%)	7 (2%)	0.63

The values are presented as no./total no. (%).

Using multivariable analysis, we examined which variables were independently associated with rehospitalization for HF. Age [HR (95% CI): 1.05 (1.01–1.1)] and hypertension [HR (95% CI): 3.25 (1.2–8.82)] were both independently associated with rehospitalization for HF. However, and most importantly, female sex *per se* was not (Table 4). As for the echocardiographic variables, only elevated LVFP during the initial hospitalization was significantly associated with rehospitalization for HF [HR (95%CI): 5.9 (2.4–14.5)], *p* < 0.001).

**Table 4:** Uni- and multivariable Cox proportional hazard analyses of rehospitalization for HF.

	Univariable		Multivariable	
	HR (95% CI)	*p*-value	HR (95% CI)	*p*-value
Age	1.09 (1.05–1.13)	<0.001	1.05 (1.01–1.1)	0.024
Female sex	3.4 (1.4–8.2)	0.006		
BMI	1.1 (0.98–1.21)	0.126		
Active smoking	0.28 (0.09–0.82)	0.02		
Hypertension	5.58 (2.17–14.39)	<0.001	3.25 (1.2–8.82)	0.02
Diabetes mellitus	2.35 (0.86–6.43)	0.095		
Dyslipidemia	1.53 (0.64–3.60)	0.332		
GFR	0.98 (0.96–0.99)	0.009		
Previous coronary artery disease	2.94 (1.22–7.08)	0.017		
Ischemia duration	1 (1–1)	0.698		
TIMI flow grade after PCI <3	1.88 (0.55–6.37)	0.312		
CK peak	1.49 (0.86–2.92)	0.20		
Initial LVEF <40	2.768 (1.15–6.68)	0.024		
Elevated LVFP at baseline	8.81 (3.71–21.0)	<0.001	5.9 (2.4–14.5)	<0.001

## Discussion

In our study, women had a twofold higher rate of HF hospitalization compared with men in the first year after anterior STEMI despite an early reperfusion. Interestingly, this increased rate is independently associated with diastolic dysfunction, age, and hypertension but not with sex.

The CIRCUS study was a recent prospective, controlled, randomized, multicenter trial in which patients with anterior STEMI underwent PCI and received optimal medical treatment as per ESC/AHA guidelines.

As already well established, women have a worse prognosis and more often develop HF than men after acute MI (15). Of note, the incidence of other major adverse cardiovascular events (MACE), including death, recurrent MI, and stroke, was not different between men and women. As opposed to previous reports, we noticed the absence between women and men of a significant difference in the time from symptom onset to PPCI and of the adjuvant medical treatment (3, 10). This increased incidence of heart failure within 1 year after STEMI was due to distinct rehospitalizations rather than an HF event occurring during the initial hospitalization.

In the era of interventional cardiology, early reperfusion therapy by PPCI prevents severe LV systolic dysfunction and persistent HF with reduced LVEF in most patients. Importantly, women and men exhibited similar areas at risk and infarct sizes, suggesting that myocardial damage *per se* cannot explain the increased incidence of rehospitalization for heart failure within the first year. Our results are in agreement with those of Kosmidou et al., who showed that with similar infarct sizes, smaller LV volumes, and better ejection fractions, women were more often rehospitalized within 1 year after acute MI than men (16). In line with this, the increased rate of rehospitalization for HF in women in our study was not associated with a more severe alteration of systolic function, as indicated by the comparable LVEF.

Epidemiological studies have shown that women represent the majority of patients with HF with preserved ejection fractions (HFpEF) (6). The risk of HFpEF increases clearly with age, hypertension, obesity, and coronary artery disease (17). Cardiac aging predisposes women to HFpEF as LV concentric remodeling and diastolic dysfunction are present to a greater degree in women than in men (18). In our study, women were older and more frequently hypertensive than men and more frequently displayed echocardiographic indices of diastolic dysfunction initially and 1 year after STEMI. To our knowledge, our study is the first one comparing the diastolic dysfunction between the sexes and studying its association with HF after a reperfused STEMI.

The multivariable analysis showed that diastolic dysfunction was independently associated with rehospitalization for HF and that initially elevated LVFP, but not baseline LVEF <40%, was associated with rehospitalization for HF. These results accord with the previous studies showing that persistent, elevated filling pressure was associated with poor outcomes both in the settings of MI and HF (19, 20). Studies have shown that LVFP first increases following coronary artery occlusion, then progressively decreases in the early hours following reperfusion (20–22).

In 666 STEMI patients treated by fibrinolysis, Kirtane et al. found that elevated left ventricular end-diastolic pressure (LVEDP) was independently associated with impaired myocardial perfusion, but also with age and female sex (23). In our study, the multivariable analysis showed that rehospitalization for heart failure was significantly associated with age, hypertension, and elevated LVFP at baseline, but not with sex.

The long-term prognostic importance of elevated filling pressure assessed invasively early after MI has been demonstrated in previous invasive studies (24, 25), particularly in terms of increase in mortality and occurrence of HF. Echocardiography assessment of LVFP correlates with invasive pressure measurements (26) and is also associated with long-term prognosis after MI. This association has been mostly studied using mitral Doppler restrictive LV filling patterns in patients with or without successful reperfusion therapy (27–29). In these studies, the initial restrictive filling pattern and age were independently associated with poor clinical outcomes, whatever the range of LVEF, including in patients with LVEF >40%.

### Limitations of the study

The main limitation of our study is its retrospective character and the fact that only 407 of the 791 patients included in the CIRCUS trial had echocardiographic data available for diastolic function assessment.

## Conclusion

Within the first year after a reperfused anterior STEMI, women presented a twofold higher rate of heart failure hospitalization compared with men, despite comparable myocardial damage and left ventricular ejection fraction. However, this increased rate was independently associated with diastolic dysfunction, age, and hypertension but not with sex. Early evaluation of diastolic dysfunction, with specific attention to women, might help prevent rehospitalization for heart failure after anterior STEMI.

## Clinical perspectives

### Competency in medical knowledge

Compared with men, women presented similar infarct sizes and ejection fractions after a reperfused anterior STEMI. However, diastolic dysfunction and subsequent rehospitalization within 1 year were more frequent in women.

### Competency in patient care

Women have a higher rate of rehospitalized for heart failure after reperfused anterior STEMI, associated with older age and more frequent hypertension and LV diastolic dysfunction. This finding raises the need for targeted therapy after STEMI, not only focused on the limitations of myocardial infarct sizes but also considering diastolic function preservation.

### Translational outlook

After a reperfused anterior STEMI, women present more diastolic dysfunction than men. The determinants of diastolic dysfunction in this context must be further investigated.

## Data Availability

The original contributions presented in the study are included in the article/**Supplementary Material**, further inquiries can be directed to the corresponding authors.
