# Supplementary material for: What underlies sex differences in heart failure onset within the first year after a first myocardial infarction?
Source: Front Cardiovasc Med. 2024 Jan 23;10:1290375. doi: 10.3389/fcvm.2023.1290375 (PMC10844509; doi:10.3389/fcvm.2023.1290375)
Supplement: Supplementary file 1 [file Table1.docx]

**Online Table 1. In hospital management**

|  | Study population  n = 407 | Female  n = 64 | Male  n = 343 | p |
| --- | --- | --- | --- | --- |
| IABP | 6/398 (1.5%) | 1/63 (1.6%) | 5/335 (1.5%) | 0.9 |
| Non invasive positive pressure ventilation | 23/398 (5.8%) | 6/63 (9.5%) | 17/335 (5.1%) | 0.23 |
| Intravenous diuretics | 35/159 (22%) | 9/31 (29%) | 26/128 (20.3%) | 0.41 |
| Inotropic agents | 4 /157 (2.5%) | 0/31 (0%) | 4/126 (3.2%) | 0.58 |
| Aspirin | 404/407 (99.3%) | 64/64 (100 %) | 340/343 (99.1%) | 0.9 |
| P2Y12 | 385/407 (94.6%) | 60/64 (93.8%) | 325/343 (94.8%) | 0.76 |
| Beta-blockers | 389/407 (95.6%) | 64/64 (100%) | 325/343 (94.8%) | 0.09 |
| Statins | 398/407 (97.8%) | 61/64 (95.3%) | 337/343 (98.3%) | 0.15 |
| ACE inhibitors or ARBs | 376/407 (92.4%) | 60/64 (93.8%) | 316/343 (92.1%) | 0.8 |
| Calcium channel blockers | 7/407 (1.7%) | 1/64 (1.6%) | 6/343 (1.7%) | 0.9 |
| Nitrates | 66/407 (16.2%) | 7/64 (10.9%) | 59/343 (17.2%) | 0.28 |

Data are presented as no. /total no. (%); median ± standard deviation; IABP: Intra-aortic Balloon Pump; ACE inhibitors: Angiotensin-Converting Enzyme inhibitors; ARBs: Angiotensin II Receptor Blockers
