# Supplementary material for: What underlies sex differences in heart failure onset within the first year after a first myocardial infarction?
Source: Front Cardiovasc Med. 2024 Jan 23;10:1290375. doi: 10.3389/fcvm.2023.1290375 (PMC10844509; doi:10.3389/fcvm.2023.1290375)
Supplement: Supplementary file 2 [file Table2.docx]

**Online Table 2. Medication at discharge**

|  | Study population  n = 407 | Female  n = 64 | Male  n = 343 | P |
| --- | --- | --- | --- | --- |
| Clopidogrel or Prasugrel or Ticagrelor | 385/407(94.6%) | 60/64 (93.8%) | 325/343(94.8%) | 0.76 |
| Aspirin | 404/407 (99.3%) | 64/64 (100 %) | 340/343(99.1%) | 0.9 |
| ACEI | 369/407 (90.7%) | 60/64 (93.8%) | 309/343(90.1%) | 0.49 |
| ARBs | 7/407 (1.7%) | 0/64 (0%) | 7/343(2%) | 0.6 |
| B-Blockers | 389/407 (95.6%) | 64/64 (100%) | 325/343(94.8%) | 0.09 |
| Calcium Channel blockers | 7/407 (1.7%) | 1/64 (1.6%) | 6/343 (1.7%) | 0.9 |
| Statin | 398/407 (97.8%) | 61/64 (95.3%) | 337/343 (98.3%) | 0.15 |
| Anti aldosterone therapy | 105/407 (25.8%) | 20/64 (31.2%) | 85/343 (24.8%) | 0.35 |
| Antivitamin K | 30/407 (7.4%) | 4/64 (6.2%) | 26/343 (7.6%) | 0.9 |
| Nitrate | 66/407 (16.2%) | 7/64 (10.9%) | 59/343 (17.2%) | 0.28 |
| Other | 341/407 (83.8%) | 60/64 (93.8%) | 281/343 (81.9%) | 0.03 |
| Diuretics | 88/407 (21.6%) | 12/64 (18.8%) | 76/343 (22.2%) | 0.65 |

Values are presented as no. /total no. (%). ACE inhibitors: Angiotensin-Converting Enzyme inhibitors; ARBs: Angiotensin II Receptor Blockers
