# Supplementary material for: What underlies sex differences in heart failure onset within the first year after a first myocardial infarction?
Source: Front Cardiovasc Med. 2024 Jan 23;10:1290375. doi: 10.3389/fcvm.2023.1290375 (PMC10844509; doi:10.3389/fcvm.2023.1290375)
Supplement: Supplementary file 3 [file Table3.docx]

**Online Table 3. Left ventricular volume echocardiographic measurements**

|  | Study population  n = 407 | Female  n = 64 | Male  n = 343 | p |
| --- | --- | --- | --- | --- |
| Baseline LVEF (%) | 46.3 ± 8.9 | 45.4 ± 9.5 | 46.4 ± 8.8 | 0.744 |
| Baseline LVEDV (ml) | 103.4 ± 29.9 | 84.7 ± 33.2 | 106.9 ± 28 | <0.001 |
| Baseline LVESV (ml) | 56.7 ± 23.3 | 47.7 ± 30.4 | 58.3 ± 21.3 | <0.001 |
| Baseline indexed LVEDV (ml/m²) | 54.2 ± 14.7 | 50.5 ± 19.4 | 54.9 ± 13.5 | <0.001 |
| Baseline indexed LVESV (ml/m²) | 29.8 ± 12.2 | 28.5 ± 18.6 | 30 ± 10.6 | 0.01 |
| One-year LVEF (%) | 50.7 ± 10.4 | 51 ± 11.9 | 50.7 ± 10.1 | 0.55 |
| One-year LVEDV (ml) | 122.2 ± 41 | 96.2 ± 32.5 | 127.1 ± 40.6 | <0.001 |
| One-year LVESV (ml) | 63.1 ± 32.5 | 49.8 ± 27.7 | 65.6 ± 32.7 | <0.001 |
| One-year indexed LVEDV (ml/m²) | 64.0 ± 20.2 | 57.3 ± 19.2 | 65.3 ± 20.2 | <0.001 |
| One-year indexed LVESV (ml/m²) | 33.1 ± 16.8 | 29.9 ± 16.9 | 33.7 ± 16.8 | 0.02 |
| LV remodeling (%) | 20 ± 30 | 20 ± 30 | 20 ± 30 | 0.297 |
| Adverse LV remodeling (no. %) | 193/403 (47.9%) | 28/63 (44.4%) | 165/340 (48.5%) | 0.64 |

Values are presented as mean± SD. LVEDV: left ventricular end diastolic volume; LVESV: left ventricular end systolic volume; LVEF: left ventricular ejection fraction; LV: left ventricule; LV volumes are indexed to body surface area; LV volumes were assessed according to the protocol during the first week following admission, and at one- year (±8 weeks); LV remodeling: (LVEDV one year-LVED baseline)/ LVEDV one year*100; Adverse left ventricular remodelling is defined by ≥15% increase of LVEDV between baseline and 1 year.
