# Supplementary material for: What underlies sex differences in heart failure onset within the first year after a first myocardial infarction?
Source: Front Cardiovasc Med. 2024 Jan 23;10:1290375. doi: 10.3389/fcvm.2023.1290375 (PMC10844509; doi:10.3389/fcvm.2023.1290375)
Supplement: Supplementary file 4 [file Table4.docx]

**Online Table 4. Echocardiographic parameters assessing diastolic function in the initial phase and at one year**

|  | **Baseline** | | **One year** | |
| --- | --- | --- | --- | --- |
|  | **Women** | **Men** | **Women** | **Men** |
| **Peak E velocity (cm/sec)** | 80.5 ± 17.6 | 70.2 ± 18.3* | 79.6 ± 19.3 | 70.1 ±16.8* |
| **Peak A velocity (cm/sec)** | 78.4 ± 28 | 64.9 ± 18.8* | 82.1 ± 26.8 | 70 ± 20* |
| **E/A ratio (no, %)** |  |  |  |  |
| <0.8 | 16 (27.6%) | 69 (21.6%) | 18 (34.6%) | 100 (31.2%) |
| 0.8-2 | 38 (65.5%) | 226 (70.8%) | 30 (57.7%) | 198 (51.9%) |
| >2 | 4 (6.9%) | 24 (7.5%) | 4 (7.7%) | 22 (6.9%) |
| **Lateral e’ velocity (cm/sec)** | 7.8 ± 2 | 8.5 ± 2.4* | 8.8 ± 2.8 | 9.5 ± 2.6 |
| **Septal e’ velocity (cm/sec)** | 6.9 ± 1.7 | 7.2 ± 1.9 | 7.2 ± 2.2 | 7.6 ± 2.0 |
| **VM DT (msec)** | 177.3 ± 48.9 | 187.6 ± 48.0 | 233.1 ± 64.6 | 221.2 ± 55.1 |
| **E/e’ ratio > 14 (no, %)** | 8 (23.5%) | 14 (8%)* | 4 (13.3%) | 9 (4.7%) |
| **LA surface (cm2)** | 18.7 ± 4.9 | 19.1 ± 4.5 | 22.4 ± 5.1 | 22.4 ± 5.4 |
| **LA max volume (mL/m²)** | 33.4 ± 12.8 | 31.7 ± 11.0 | 43.1 ± 14.0 | 39.2 ± 13.8 |
| **TR systolic jet velocity (m/s)** | 2.7 ± 0.4 | 2.5 ± 0.4* | 2.6 ± 0.5 | 2.4 ± 0.5* |

VM DT: mitral deceleration time; LA: left atrium, TR: tricuspid regurgitation; *: p <0.05 vs. women
